# Introduced ascidians harbor highly diverse and host-specific symbiotic microbial assemblages

**DOI:** 10.1038/s41598-017-11441-4

**Published:** 2017-09-08

**Authors:** James S. Evans, Patrick M. Erwin, Noa Shenkar, Susanna López-Legentil

**Affiliations:** 1 Department of Biology & Marine Biology, and Center for Marine Science, University of North Carolina Wilmington, 5600 Marvin K. Moss Lane, Wilmington, NC 28409 United States of America; 2 Department of Zoology, and The Steinhardt Museum of Natural History, Israel National Center for Biodiversity Studies, Tel-Aviv University, Tel Aviv, 69978 Israel

## Abstract

Many ascidian species have experienced worldwide introductions, exhibiting remarkable success in crossing geographic borders and adapting to local environmental conditions. To investigate the potential role of microbial symbionts in these introductions, we examined the microbial communities of three ascidian species common in North Carolina harbors. Replicate samples of the globally introduced species *Distaplia bermudensis*, *Polyandrocarpa anguinea*, and *P. zorritensis* (*n* = 5), and ambient seawater (*n* = 4), were collected in Wrightsville Beach, NC. Microbial communities were characterized by next-generation (Illumina) sequencing of partial (V4) 16S rRNA gene sequences. Ascidians hosted diverse symbiont communities, consisting of 5,696 unique microbial OTUs (at 97% sequenced identity) from 44 bacterial and three archaeal phyla. Permutational multivariate analyses of variance revealed clear differentiation of ascidian symbionts compared to seawater bacterioplankton, and distinct microbial communities inhabiting each ascidian species. 103 universal core OTUs (present in all ascidian replicates) were identified, including taxa previously described in marine invertebrate microbiomes with possible links to ammonia-oxidization, denitrification, pathogenesis, and heavy-metal processing. These results suggest ascidian microbial symbionts exhibit a high degree of host-specificity, forming intimate associations that may contribute to host adaptation to new environments via expanded tolerance thresholds and enhanced holobiont function.

## Introduction

Modern society is founded upon the rapid transgression of people and goods across geographic borders; however, this globalization has come at an ecological cost: the introduction of nonnative species^1^. The marine environment has a high potential for alien colonization, as there are few physical barriers to impede the spread of invasive species. Consequently, the invasion rate of marine ecosystems, while already high, appears to be rapidly increasing^2^. In most cases, complete eradication of nonnative species from an invaded region is impossible^3^. Prevention is the most effective and least costly means of invasive species control^4^, but predicting which species will prove successful as invaders and what locations will be susceptible to nonnative introductions has proved remarkably difficult^5^. In locations where preventative measures have already failed, management of invasive populations is the most feasible approach^3^, though the effectiveness of these techniques varies^5^. Generally, the most effective management strategies are those that focus on the ecosystem as a whole, rather than on individual invasive species^5^ or specific introduction vectors^4^. This requires early detection^3,5^, in addition to a comprehensive knowledge of the biology of the invading organism^5^ and its interactions with the ecosystem^4,5^. Consequently, a complete understanding of the physiology and ecology of invading organisms, as well as the environmental factors that allow their establishment, is critical to the successful management of marine invasions.

Ascidians, commonly called sea-squirts, are sessile marine invertebrates of the phylum Chordata. Ascidian larvae are short-lived, non-feeding, and capable of swimming only short distances, while adults are sessile, benthic filter feeders^6^. Yet despite their limited dispersal potential, many ascidian species have been successfully introduced around the world, and rank among the taxa with the highest recorded numbers of introduced species^7,8^. This suggests that ascidians are likely introduced to new habitats via passive transport, either as fouling organisms attached to the hulls of ships^6,9–11^ or, in rare cases, as larvae within ballast water^12^. Introduced ascidians have been initially observed confined to artificial substrates previously unoccupied and rarely colonized by native inhabitants^13^. After a few adults are established, other available artificial surfaces, such as boat hulls or the pilings and floating docks of marinas and harbors, are progressively colonized by ascidian larvae^14^.

Harbor systems frequently exhibit polluted conditions, yet many nonnative ascidians persist and even thrive within these habitats, exhibiting tolerance to sewage, surface runoff ^7^, and toxic heavy metals^15^. Additionally, once introduced, many ascidians possess a broad range of environmental tolerances that enhance long-term survival, including resistance to wide fluctuations in temperature^7,15,16^, salinity^15,16^, and both organic^13,17^ and inorganic pollution^15^. Many introduced ascidians are characterized by high growth rates and high fecundity, leading to increases in local abundance^14^. Once established, ascidians become fierce competitors for space, either by simply overgrowing surrounding benthic organisms^18^, or by producing allelopathic metabolites to inhibit growth in nearby competitors^19^ or reduce the larval recruitment of other species^20^. Over the longer term, this results in a significant increase in ascidian populations within the afflicted region, often at the expense of the native benthos^21^.

The ascidian tunic, a cellulosic outer coating that surrounds the animal, may also contribute to the successful establishment of introduced ascidian species. Ascidians are remarkable for their abundance within systems in which predation is notoriously heavy^22^. This resistance to predation may be attributed to physical defenses of the tunic, such as tunic toughness^23^, or to chemical defenses, such as acidity or the production of secondary metabolites to deter feeding^22,24–26^. Of these putative defense mechanisms, however, secondary metabolite production has been suggested as the primary means of predation avoidance^26^. Additional studies have indicated that symbiotic bacteria, living within the tunic of ascidians, may be responsible for the production of at least some of these defensive secondary metabolites^27^.

In addition to secondary metabolite production, many of the unique biochemical capabilities of marine organisms can be traced to prokaryotic symbionts (e.g. bioluminescence)^28^. In ascidians, symbiotic relationships with bacteria and photosynthetic unicellular algae have been described, which may represent mutualistic associations that benefit both symbiont (protection) and host (nutrition and defense)^29,30^. Further, many symbiont functions (e.g. nitrification) are thought to translate into enhanced holobiont functionality, thus potentially contributing to the survival of the ascidian host^30,31^. Studies using culture-independent, DNA sequence-based techniques have revealed a high degree of microbial diversity in ascidians that is host-specific and appears to remain temporally consistent^32–34^.

The functionality of diverse ascidian microbiomes may play an especially important role for introduced ascidians that must adapt to new environments. For example, symbiont-mediated processing of pollutants or innocuous bioaccumulation of heavy metals that characterize harbor waters may allow for the establishment of a founder population within a new habitat. To date however, the role that microbial symbionts play in the successful establishment of introduced ascidians remains largely unknown, as only a couple of studies have described the diverse symbiont communities present in introduced ascidian hosts^35,36^. In this study, we examined the microbial communities associated with three globally introduced ascidian species that are common in North Carolina harbors: *Polyandrocarpa zorritensis*, *P. anguinea*, and *Distaplia bermudensis*. We utilized next-generation sequencing of the bacterial/archaeal 16S rRNA gene to characterize the symbiont diversity and host-specificity, and to gain insight into the potential contribution of these symbionts to the successful establishment of these species in North Carolina waters.

## Results

### Symbiotic microbial community composition and diversity

**Figure 1:**
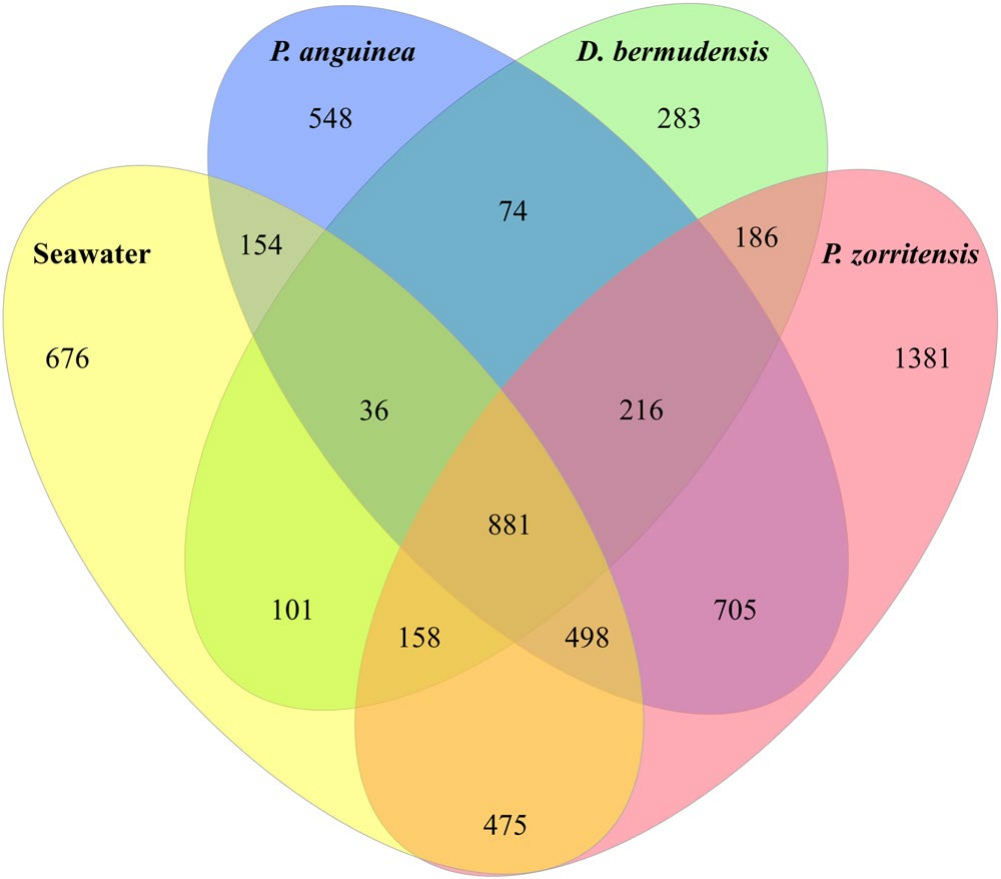
Venn diagram depicting OTU richness and overlap in microbial communities of *Distaplia bermudensis* (green), *Polyandrocarpa anguinea* (blue), *Polyandrocarpa zorritensis* (red), and ambient seawater (yellow). Total OTU richness was 6,372 OTUs among the four sources.

**Figure 2:**
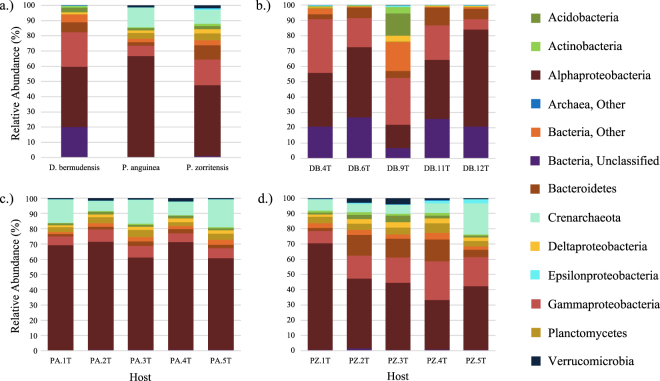
Microbial community composition averaged for the three ascidian species (**a**), and for each replicate sample of the ascidians *D. bermudensis* (**b**), *P. anguinea* (**c**), and *P. zorritensis* (**d**). Phylum-level classifications are shown, except for *Proteobacteria* which are divided into major classes.

In total, 6,372 OTUs were recovered from *Distaplia bermudensis* (*n* = 1,935), *Polyandrocarpa anguinea* (*n* = 3,112), *P. zorritensis* (*n* = 4,500), and ambient seawater (*n* = 2,979, Fig. 1). Nearly half of these OTUs (45%, *n* = 2,888) were exclusively detected in one source: seawater (*n* = 676), *D. bermudensis* (*n* = 283), *P. anguinea* (*n* = 548), or *P. zorritensis* (*n* = 1,381) (Fig. 1). 881 OTUs were shared by all four sources, 1,097 OTUs were shared by all three ascidian species (i.e. universal ascidian symbionts), and 3,393 OTUs were recovered from one or more ascidian species and not present in the seawater (Fig. 1). Ascidian-sourced OTUs spanned 44 bacterial and three archaeal phyla (Euryarchaeota, Crenarchaeota, and Parvarchaeota), with Euryarchaeota and Crenarchaeota represented in all three ascidian species, though relative abundances varied among the different sources (Fig. 2a). Parvarchaeota was detected only in the two Polyandrocarpa species, and only in low concentrations. Bacterial phyla likewise differed in relative abundance among the three species investigated (Fig. 2a). Microbial communities in D. bermudensis included 32 bacterial phyla and were dominated by Alphaproteobacteria (38%), Gammaproteobacteria (22%), unclassified bacteria (19%), and Bacteroidetes (6%; Fig. 2b). Symbiont communities in P. anguinea included 39 bacterial phyla and were dominated by Alphaproteobacteria (66%) and Gammaproteobacteria (7%), as well as Crenarchaeota (13%; Fig. 2c). Microbial communities in P. zorritensis included 42 bacterial phyla and were dominated by Alphaproteobacteria (47%), Gammaproteobacteria (17%), Bacteroidetes (9%), and Planctomycetes (5%), as well as Crenarchaeota (9%; Fig. 2d).

The diversity metrics OTU richness (S), the Shannon-Weaver diversity index (H′), and the Chao1 index were all significantly different (*P* < 0.05) among the three ascidian species for the overall data partition (Supplementary Table S1). Pairwise comparisons for the overall data partition indicated that *P. zorritensis* exhibited significantly greater observed (S) and expected (Chao1) microbial richness than *P. anguinea*, which exhibited a significantly greater richness than *D. bermudensis* (Supplementary Table S1). *P. zorritensis* exhibited a significantly greater Shannon diversity (H′) than *D. bermudensis*, but was not significantly different from *P. anguinea* at the overall level (Supplementary Table S1). No significant differences in the evenness or Simpson diversity indices were detected among the microbial communities of the three species at the overall level (Supplementary Table S1).

### Symbiotic microbial community structure

**Figure 3:**
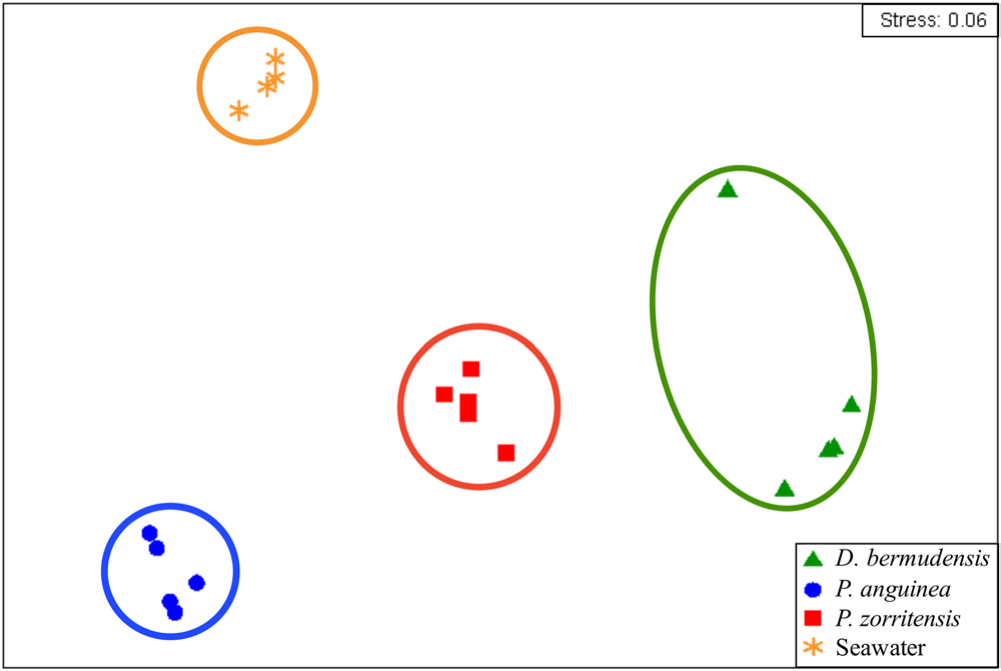
Multi-dimensional scaling plot based on Bray-Curtis similarity of microbial communities in *Distaplia bermudensis* (green triangles), *P. anguinea* (blue circles), *P. zorritensis* (red squares), and seawater (yellow stars). Circles encompass replicate samples within each source and indicate a high degree of host-specificity.

An assessment of microbial community structure among the different sources revealed that communities clustered in response to source (Fig. 3), with significant differences between the microbial communities of *D. bermudensis*, *P. anguinea*, and *P. zorritensis*, as well as ambient seawater (PERMANOVA, *P* < 0.01), and 78% of the community structure variation explained by source. Pairwise comparisons revealed significant differences (PERMANOVA, *P* < 0.015) between microbial communities of each host source, and ambient seawater. Within each source, seawater exhibited less variability in microbial community structure among replicates (average similarity = 79.0%) compared to the microbiomes of *D. bermudensis*, *P. anguinea*, and *P. zorritensis* (average similarities = 55.9%, 64.0%, and 57.3%, respectively); however, no significant differences in dispersion were detected among sources (PERMDISP, *P* = 0.221).

**Figure 4:**
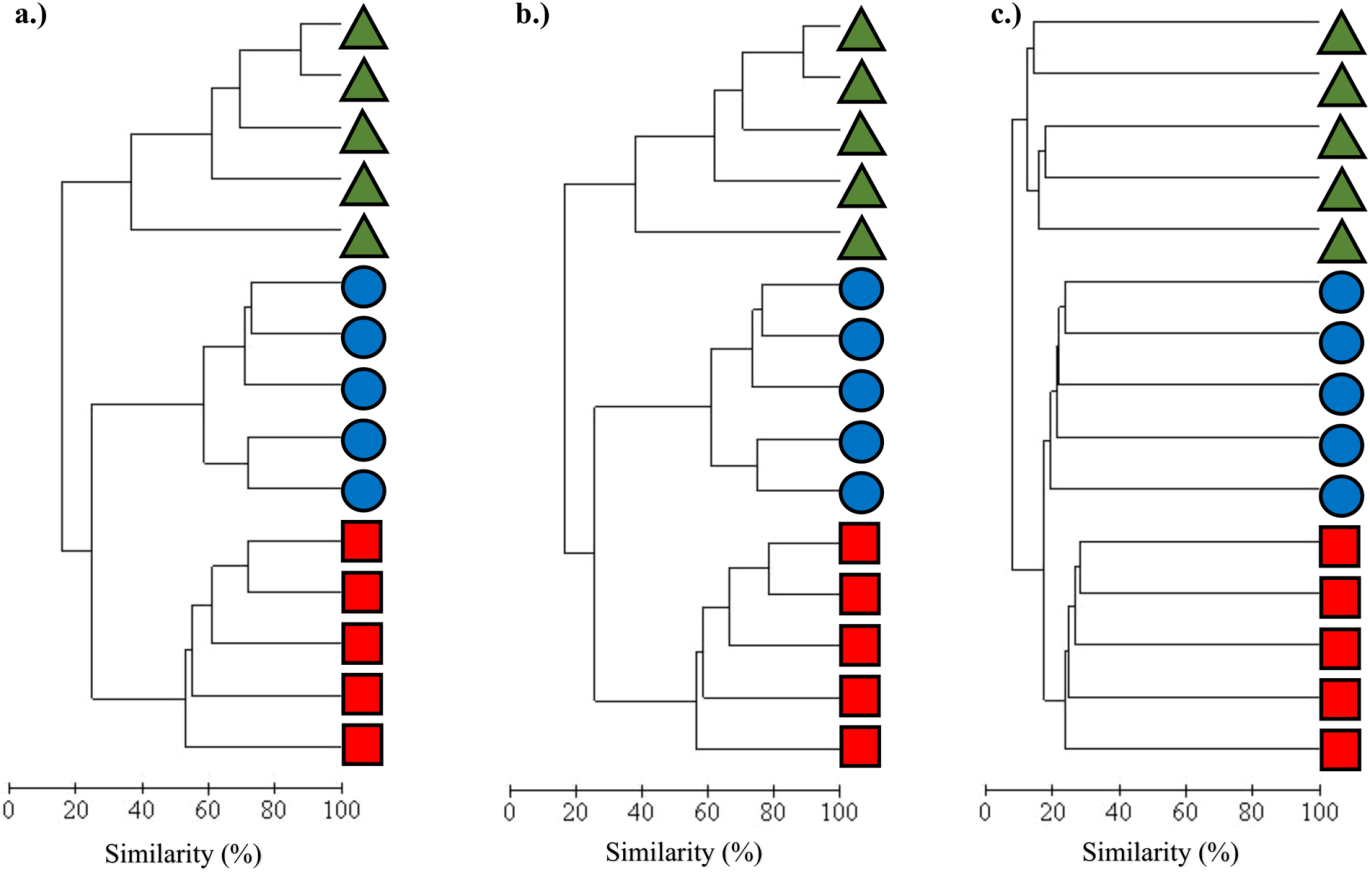
Cluster dendrograms based on Bray-Curtis similarity of microbial communities in *D. bermudensis* (green triangles), *P. anguinea* (blue circles), and *P. zorritensis* (red squares), showing results for the overall (**a**), abundant (**b**), and rare (**c**) OTU data partitions.

Examining the ascidian microbial communities exclusively, community structure was again significantly different among species (PERMANOVA, *P* < 0.01), with host species explaining 72.6% of the variation in overall community structure. Partitioning of the ascidian microbial community data into abundant (>0.1% relative abundance) and rare (≤0.1%) components resulted in 751 abundant OTUs that accounted for 96.9% of the total sequences and 4,945 rare OTUs that accounted for 3.1% of the total sequences, post singleton removal. Both the abundant and rare OTU data partitions indicated significant differences in microbial community structure (PERMANOVA, *P* < 0.01), with source explaining 75% of the variation in the abundant community structure, but only 18.6% of the variation in the rare community structure. Multiple pairwise comparisons revealed significant differences between the microbial communities of all ascidian host sources, for all three data partitions (Table 1). The permutational multivariate analysis of dispersion across groups was not significant for either the overall or abundant OTU data partitions (PERMDISP, *P* = 0.86 and *P* = 0.818, respectively), but was significant for the rare OTU data partition (PERMDISP, *P* < 0.01), with all subsequent pairwise comparisons likewise proving significant (Table 1). Additionally, for the rare OTU data partition, greater dissimilarity was observed between replicates within each species (Fig. 4c) compared to the overall or abundant data partitions (Fig. 4a,b).

**Table 1:** Pairwise statistical comparisons of microbial community structure (PERMANOVA) and dispersion (PERMDISP) in *Distaplia bermudensis*, *Polyandrocarpa anguinea*, and *P. zorritensis* at overall, abundant, and rare partition levels*.

	Overall				Abundant				Rare			
	PERMANOVA		PERMDISP		PERMANOVA		PERMDISP		PERMANOVA		PERMDISP	
Pairwise comparison	*t*	*p*	*t*	*p*	*t*	*p*	*t*	*p*	*t*	*p*	*t*	*p*
*D. bermudensis*	4.39	0.007*	0.76	0.742	4.57	0.006*	0.92	0.602	1.48	0.009*	4.72	0.010*
*P. anguinea*												
*D. bermudensis*	3.21	0.007*	0.13	0.945	3.37	0.005*	0.44	0.832	1.57	0.005*	7.70	0.008*
*P. zorritensis*												
*P. anguinea*	3.68	0.014*	1.29	0.279	4.02	0.011*	0.84	0.489	1.32	0.007*	2.79	0.008*
*P. zorritensis*												

*Significantly different following B-Y correction.

The congeneric species *Polyandrocarpa zorritensis* and *P. anguinea* exhibited more similar microbiomes compared to the distantly related host *D. bermudensis* (Fig. 4a–c), though the differences observed between these closely related ascidians were still significant (Table 1). *P. anguinea* and *D. bermudensis* were the most dissimilar, with an average dissimilarity of 91.9, 91.9 and 92.1% for the overall, abundant OTU and rare OTU datasets, respectively. *D. bermudensis* and *P. zorritensis* had an average dissimilarity of 76.5, 75.4 and 91.8% across the three dataset partitions respectively, while *P. anguinea* and *P. zorritensis* had an average dissimilarity of 75.1, 74.4 and 82.5%. SIMPER analyses identified the top OTUs responsible for the observed dissimilarity between ascidian microbiomes (Table 2). For the overall microbial community and the abundant OTU data partition, a small number of symbiont OTUs (four or five) contributed to the majority (>50%) of the observed dissimilarity among hosts (Table 2). In contrast, the differences between the rare microbial communities of the three species or sources were driven by the presence or absence of a great number of microbial taxa (569 to 831 OTUs).

**Table 2:** Taxonomic classification and relative abundance of symbiont OTUs contributing to the observed dissimilarity in microbial community structure (50% cumulative, SIMPER analyses) between *D. bermudensis* (DB), *P. anguinea* (PA), and *P. zorritensis* (PZ).

Comparison	Dissimilarity (%)	OTU	Phylum	Lowest Taxonomy*	Rel. Abund. (%)		Contribution to Dissimilarity (%)
					DB	PA	
DB, PA	91.94	2	Proteobacteria	S. *Rhodobium orientis*	0.5	33.8	18.15
		3	unclassified	K. Bacteria	19.6	0.1	10.60
		1	Proteobacteria	F. Rhodobacteraceae	19.1	1.3	9.66
		5	Proteobacteria	F. Rhodobacteraceae	17.6	0.8	9.12
		7	Proteobacteria	G. *Nitrosopumilus*	15.6	0.2	8.35
					**DB**	**PZ**	
DB, PZ	76.45	3	unclassified	K. Bacteria	19.6	0.2	12.71
		1	Proteobacteria	F. Rhodobacteraceae	19.1	29.5	12.41
		5	Proteobacteria	F. Endozoicimonaceae	17.6	1.0	10.86
		7	Proteobacteria	G. *Nitrosopumilus*	15.6	0.2	10.07
		4	Crenarchaeota	G. *Nitrosopumilus*	0.1	8.9	5.81
					**PA**	**PZ**	
PA, PZ	75.08	2	Proteobacteria	S. *Rhodobium orientis*	33.8	0.5	22.18
		1	Proteobacteria	F. Rhodobacteraceae	1.3	29.5	18.78
		8	Proteobacteria	F. Rhodobacteraceae	12.7	1.0	8.07
		9	Proteobacteria	F. Rhodobacteraceae	6.6	0.4	4.22

*K kingdom, *P* phylum, *C* class, *O* order, *F* family, *G* genus, *S* species.

### Core symbiont community composition and diversity

Core symbiont communities were identified for each ascidian species (i.e. OTUs shared by all five replicates) and for all ascidian hosts (i.e. OTUs shared by all five replicates from all three species). The core community in *D. bermudensis* consisted of 147 OTUs that comprised 93.3% of all sequences, with the top 15 most prevalent core OTUs accounting for the vast majority (84.8%) of all sequences (Supplementary Table S2). The core microbial community in *P. anguinea* consisted of 355 OTUs that comprised 90.4% of all sequences, with the top 15 most prevalent core OTUs comprising 75.8% of all sequences (Supplementary Table S2). The core microbial community in *P. zorritensis* consisted of 552 OTUs that comprised 84.8% of all sequences, with the top 15 most prevalent core OTUs accounting for 54.9% of all sequences (Supplementary Table S2). Across all ascidian hosts, 103 universal core OTUs were identified. Although the relative abundance of these universal core OTUs varied among hosts (Table 3), these 103 OTUs represented just 1.81% of all detected ascidian OTUs, but 79.1% of all sequences. In fact, the top 15 of these 103 OTUs represented 68.9% of all sequences and corresponded to one archaeal (Crenarchaeota) and 14 bacterial symbionts. Of the bacterial OTUs, seven were affiliated with Alphaproteobacteria, four with Gammaproteobacteria, two with Bacteroidetes, and one was unclassified at the phylum level (Table 3). This one unclassified OTU represented 98.3% of all unclassified bacterial sequences in *D. bermudensis* and 94.0% of unclassified sequences for all three species together.

**Table 3:** Relative abundance, closest BLASTn sequence match, and taxonomic classification of universal core OTUs in *D. bermudensis* (DB), *P. anguinea* (PA) and *P. zorritensis* (PZ).

OTU	Rel. Abund. (%)			BLASTn Match			Taxonomy	
	DB	PA	PZ	Acc. No.	ID (%)	Source	Phylum	Lowest Classification
1	19.1	1.3	29.5	GU066483	98.8	Marine Biofilm	Proteobacteria	F. Rhodobacteraceae
2	0.5	33.8	0.5	HM178732	98.4	Marine Sediment	Proteobacteria	S. *Rhodobium orientis*
3	19.6	0.1	0.2	AM997291	92.0	Marine Sediment	unclassified	K. Bacteria
4	0.1	10.9	8.9	KJ504303	100	Sponge	Crenarchaeota	G. *Nitrosopumilus*
5	17.6	0.8	1.0	HM768514	99.6	Coral	Proteobacteria	F. Endozoicimonaceae
7	15.6	0.2	0.2	JQ726961	92.8	Marine Biofilm	Proteobacteria	G. *Nitrosopumilus*
8	0.4	12.7	1.0	FJ203573	100	Coral	Proteobacteria	F. Rhodobacteraceae
9	0.1	6.6	0.4	JF261902	100	Marine Biofilm	Bacteroidetes	F. Rhodobacteraceae
10	6.5	<0.1	0.1	DQ884168	90.8	Ascidian	Bacteroidetes	G. *Reichenbachiella*
11	0.1	6.2	0.2	EF123355	97	Coral	Proteobacteria	G. *Hyphomonas*
6	0.4	0.6	2.8	KT880280	100	Seawater	Proteobacteria	F. Rhodobacteraceae
16	<0.1	<0.1	2.8	AY499775	91.2	Marine Sediment	Bacteroidetes	P. Bacteroidetes
17	<0.1	<0.1	2.2	GQ412848	99	Marine Sediment	Proteobacteria	G. *nsmpvI18*
18	0.9	0.2	0.8	KR075025	100	Prawn	Proteobacteria	S. *Vibrio shilonii*
22	0.1	0.4	1.1	JF948094	100	Marine Biofilm	Proteobacteria	F. Piscirickettsiaceae

*K* kingdom, *P* phylum, *C* class, *O* order, *F* family, *G* genus, *S* species.

## Discussion

With the rapid advances in sequencing technology, our understanding of the complex interactions between microbial symbionts and their hosts continues to expand. Here, we examined the microbial communities of three widely introduced ascidian species, as well as those of the surrounding ambient seawater, as a first step in understanding the role that microbial symbionts may play in the successful establishment of these ascidian species in North Carolina. The microbial communities of *D. bermudensis*, *P*. *anguinea*, and *P*. *zorritensis* exhibited significant differences in both community structure and diversity. Microbial communities in the three species were also clearly different from those of the surrounding seawater, as previously reported for other ascidian species^32,34,36^. Over half (53.2%) of all OTUs obtained herein were present only in ascidian hosts and not detected in ambient seawater. Comparisons of microbial community structure among the three ascidian species revealed a high degree of host-specificity, with significant differences in microbial community structure detected among all hosts. Interestingly, the microbiomes of the two congeneric ascidian species were more structurally similar to one another than to *D. bermudensis*, providing further support for the host-specificity of the ascidian-microbe interactions described herein. Within each ascidian species, broad taxonomic comparisons (phylum-level) indicated a relatively conserved symbiont composition across replicates. At the OTU-level, replicates within the same species exhibited at least 55% average similarity for both the overall and abundant OTU data partitions. Intra-specific variation was explained primarily by the presence or absence of numerous rare OTUs, for which the average similarity within hosts ranged from 13.7% (*D. bermudensis*) to 25.1% (*P. zorritensis*).

The high degree of host-specificity in ascidian microbiomes observed herein further supports previous findings indicating that each ascidian tunic possesses unique internal features or physiological characteristics that provide favorable conditions (i.e. microniches) for supporting distinct microbial communities^32,37^. Both vertical and horizontal symbiont transmission may be involved in the establishment and maintenance of these distinct microbiomes^31^. A high degree of host-specificity indicates that some degree of vertical transmission occurs in colonial ascidians, as further supported by microscopic observations of bacteria in ascidian larvae^34,38^. Ascidians also appear to selectively accumulate specific microbes from the surrounding seawater, as rare seawater microbes have been shown to be enriched in ascidian tunics^31^. More relevant to this particular study, the ascidian *Polyandrocarpa zorritensis* has been shown to accumulate pollution indicator microbes directly from seawater^17^, suggesting horizontal transmission may also play a role in construction of the microbiome of introduced ascidians. Indeed, in the current study, most universal ascidian symbiont OTUs (80.3%) were also detected in the seawater, with some of these OTUs matching identically (100% pairwise identity) to sequences previously reported from seawater samples or sponge tissue within the same region^39^. Given the significant structural differences between the microbial communities of the four sources, these similarities are especially striking. Taken together, our findings and those of previous studies suggest that microbial symbionts within ascidian species are sourced both vertically via transmission from parent colonies and horizontally through uptake from the local environment, supporting an environmentally “leaky” model of vertical transmission in ascidians that has previously been proposed for marine sponges and mussels^40^. Such a strategy could greatly benefit ascidians introduced into a new habitat by arriving seeded with beneficial symbionts (e.g. those contributing to host primary metabolism), yet also being capable of acquiring locally-adapted (e.g. pollution tolerant) symbionts. Thus, both the microbial inheritance of each introduced ascidian and the microbial residents of each susceptible habitat may represent important factors in the successful establishment of introduced species.

The host-specific core communities of all three ascidian species were comprised of a small number of OTUs with disproportionate abundances, accounting for the vast majority (85 to 93%) of all sequences obtained for *D. bermudensis*, *P. anguinea*, and *P. zorritensis*. A prior study of the microbiome in the globally introduced ascidian *Styela plicata* found a similarly disproportionate representation of a few core OTUs^35^. The existence of core OTUs suggests that these particular symbionts may play an important role in the survival of a particular host species, especially OTUs with high relative abundances, as they have been linked to increased host functionality in marine invertebrates^41^. However, it should be noted that the rare microbiome has been shown in some cases to dominate certain processes, such as nutrient cycling, despite low microbial abundances^42–44^, and thus the functional implications of rare symbionts within the ascidian microbiome should not be disregarded. Microbial symbionts may contribute to ascidian host survival in several ways, including supplementing host nutrition (e.g. *Prochloron* in some didemnid species^45^), providing defense through the production of secondary metabolites^24,27,30^, or providing antifouling capabilities^20,46^. Microbial symbionts have been linked to the success of invasive species within a terrestrial framework, with the presence of specific endosymbionts being directly related to increases in invasive plant biomass^47^. Whether microbial symbionts play similar roles in facilitating successful marine invasions is still poorly understood, and the potential benefits of those symbionts to the holobiont are not well-characterized.

The identification of 103 universal core OTUs (i.e. detected in all replicates of all species) herein is especially interesting in the context of invasiveness potential. The top 15 universal core OTUs varied in relative abundance among hosts, yet accounted for a large proportion of all sequences obtained, with a potentially dominating effect on symbiotic functions within introduced ascidians. Universal core OTUs were affiliated with archaeal and bacterial taxa that exhibit functional capabilities of potential benefit to the holobiont, including nitrogen cycling, protection against UV radiation, and heavy metal processing. The most prevalent archaeal symbiont in the universal core community was assigned to the genus *Nitrosopumilus*, an ammonia-oxidizing archaeal lineage^48^ that has been described in association with other ascidian hosts^31,32^. Ascidians expel the majority of their nitrogenous waste in the form of ammonia^49^, and high concentrations of ammonia-rich waste within the tunic may support growth of *Nitrosopumilus* symbionts. In return, ammonia-oxidizers may benefit ascidian hosts by processing and removing nitrogenous waste. Similarly, two lineages of proteobacterial denitrifiers (*Rhodobium orientis*^50^ and *Novispirillum* sp.^51^) were identified as top universal core OTUs within the three ascidian species. Denitrification involves the microbial reduction of nitrate and may also contribute to successful ascidian introductions within polluted harbor systems by increasing the tolerance threshold of the holobiont to higher nitrogen pollution levels. Harbor systems, in which ascidians are most commonly introduced, are notoriously polluted due to their close proximity to anthropogenic development, contamination from high boat traffic (e.g. fuel combustion and spills^52^, leaching of anti-fouling hull paints^53^), and their relative isolation from the open ocean. As a result, pollutants become concentrated within harbor systems, including toxic heavy metals^54^, hydrocarbons^54^, and nitrogenous pollution from sewage effluence^55^. This reduced circulation and increased pollution in harbors has been linked to permanent changes in ascidian community structure^56,57^, and localized increases in introduced ascidian species have been observed in harbor systems at the apparent expense of native ascidian populations^7^. Additional studies targeting nitrifying and denitrifying symbiotic microbial guilds are needed to assess their contribution to the invasiveness potential of introduced ascidian species in polluted harbor environments, as well as the degree to which ammonia oxidization and denitrification capabilities may enhance holobiont metabolism. Notably, the simultaneous occurrence of symbiont taxa capable of aerobic and anaerobic nitrogen transformation pathways suggests that these symbionts may occur in distinct regions of the inner tunic, or are separately maintained by anoxic and normoxic conditions generated by the process of filter feeding, as has been suggested for marine sponges^58^.

In addition to putative roles in nitrogen cycling, universal core OTUs in introduced ascidians also matched to bacterial taxa capable of carotenoid production and heavy metal processing. Two universal core symbionts, *R. orientis* and *Reichenbachiella* sp., produce carotenoids^50,59^, which have been suggested to provide UV protection in other marine invertebrate hosts^60^ and could be especially critical for survival in shallow harbor environments. *Vibrio shilonii*, another universal core symbiont, may provide heavy metal processing capabilities to ascidian hosts, as symbionts in the genus *Vibrio* found within marine invertebrates have shown resistance to heavy metal toxicity^61^. More specifically, a comparative genomic analysis of *Vibrio* spp. identified a gene coding for the copper chaperone protein CopZ within *V. shilonii*, suggesting this particular symbiont could metabolize copper^62^. This heavy metal processing capability could help explain how introduced ascidians continue to thrive in polluted harbor regions, even as native species disappear^7,8^. However, the extent to which these capabilities would affect the holobiont fitness and long-term colonization success is unclear. In addition, *V. shilonii* (also called *V. mediterranei*^63,64^) is a known coral pathogen that has been linked to coral bleaching events^65^. The presence of documented invertebrate pathogens as core symbionts in introduced ascidians provides preliminary evidence for an additional negative ecological impact of species introductions to native ecosystems. Putative pathogenic microbial symbionts have been previously described within other invertebrates^66,67^, including ascidians^68^, suggesting that invertebrate microbiomes may act as reservoirs for those pathogens. Indeed, *V. shilonii* was able to survive lethal winter temperatures within invertebrate hosts, which subsequently served as pathogen vectors^66^. Clearly, the full ecological impacts of species introductions on ecosystem health are not well understood and merit further investigation. As global changes in temperature regimes continue to favor introduced species to the detriment of native ones^69^, including ascidians^70^, a greater understanding of microbiome contributions to invasiveness potential and ecosystem damage will help clarify pathways for invasion success and prevention.

## Materials and Methods

### Sample collection and DNA extraction

Three species were targeted in this study: *Polyandrocarpa zorritensis*, *P. anguinea*, and *Distaplia bermudensis*. All three species are considered introduced in North Carolina^71^. *P*. *zorritensis* was first described by Van Name in Peru^72^ and exhibits a worldwide distribution, including Panama^73^, the southeastern United States^71^, Italy^74^, and Spain^75^. *P. anguinea* was first described by Sluiter in Mozambique^76^, and has since been described in Brazil^77^, Panama^73^ and the southeastern US^71^. *D. bermudensis* was first described by Van Name in Bermuda^78^, and has since been observed in Puerto Rico^79^, the southeastern US^71,79^, Spain^80^, and Italy^81^.

Ascidian and ambient seawater samples were collected at two nearby sites (<0.15 km apart) in Wrightsville Beach, NC. Replicates of *D. bermudensis* (*n* = 5) were collected in June 2015 from the Bridge Tender Marina (34°13′06″N 77°48′47″W). Replicates of *P. anguinea* (*n* = 5) and ambient seawater (*n* = 1) were collected in September 2015 from Wrightsville Beach Marina (34°12′59.6″N 77°48′45.7″W). Replicates of *P. zorritensis* (*n* = 5) and ambient seawater (*n* = 3) were collected in October 2015 from the Wrightsville Beach Marina site. Ascidian identifications in the field were based on morphological characteristics, and confirmed in the lab via barcoding of a fragment of the mitochondrial gene cytochrome *c* oxidase subunit I (COI; see below). All samples were collected at <1 m depth and at least 5 m apart to minimize the probability of sampling clones. Samples were housed separately in Whirl-Pak bags containing ambient seawater. Once in the laboratory (less than 10 km away from the sampling sites), each ascidian sample was dissected in half. One half was immediately fixed in 10% formalin and filtered seawater for morphological analysis, and stored at room temperature. The other half of each colony was rinsed with filtered seawater and stored in absolute ethanol at −20 °C for molecular analysis. Ambient seawater samples (500-ml each) were concentrated onto 0.2-μm filters and immediately frozen at −80 °C until analysis. Prior to DNA extraction, ethanol-preserved ascidian samples were dissected under a stereomicroscope into zooid and tunic fractions for host barcoding and symbiont characterization, respectively. DNA extractions of the ascidian zooids, inner tunics (i.e., tunic tissue not in contact with either the surrounding seawater or the zooids), and seawater filters were performed with the DNeasy Blood and Tissue kit (Qiagen).

### Ascidian genetic barcoding

To confirm morphological identifications of collected ascidians, a ca. 600-bp segment of the mitochondrial COI gene was amplified from zooid extractions using the universal LCO1490 and HCO2198 primers^82^ or the ascidian-specific primers Tun_forward and Tun_Reverse2^83^. PCR amplifications were conducted with a total volume of 25 μl, including 5 pmol of each primer, 2X MyTaq HS Red Mix (Bioline), and 1 μl (ca. 10 ng) of template DNA. The thermocycler program consisted of an initial denaturation at 95 °C for 1 min; 35 cycles of 95 °C for 15 s, 45 °C for 15 s, and 72 °C for 10 s; and a final extension at 72 °C for 1 min. Sequence reactions were conducted with BigDye Terminator v. 3.1 (Applied Biosystems) and the same forward and reverse primers utilized in the initial amplifications. PCR products were purified via isopropanol precipitation or BigDye Xterminator (Applied Biosystems) and sequenced on an ABI 3130xl genetic analyzer (Applied Biosystems) or an AB 3500 genetic analyzer (Applied Biosystems), both available at UNCW Center for Marine Science. Raw sequence reads were processed in Geneious version 8.02^84^ by aligning forward and reverse reads to create consensus sequences. These consensus sequences were compared to sequences within the GenBank database using a nucleotide-nucleotide BLAST search^85^ (BLASTn) and archived in GenBank under the accession numbers MF034529 to MF034540.

### Next-generation sequencing of microbial symbionts

To characterize microbial communities in ascidian tunics and seawater samples, a ca. 300 bp fragment (V4 region) of the 16S rRNA gene was amplified and sequenced from tunic and seawater extracts using the universal bacterial/archaeal forward primer 515f and reverse primer 806r^86^. Initial PCR amplifications were used to confirm the viability of DNA extractions, with a thermocycler program consisting of an initial denaturation at 95 °C for 2 min; 35 cycles of 95 °C for 15 s, 50 °C for 15 s, and 72 °C for 20 s; and a final extension at 72 °C for 2 min. DNA extracts were sent to Molecular Research LP for amplification, library construction and multiplexed sequencing of partial 16S rRNA gene sequences with the 515f and 806r primers on an Illumina MiSeq platform. Raw sequence data were deposited in the Sequence Read Archive of NCBI (accession no. SRP106072).

### Next-generation sequence data processing

Raw sequences were processed in the mothur software package^87^ following a modified version of the Illumina MiSeq SOP pipeline^88^ as described previously^39^. Briefly, raw sequences were quality-filtered and aligned to the SILVA reference database (v119), putative chimeric sequences were removed via self-reference searching with UChime^89^, sequences were classified using a naive Bayesian classifier and bootstrap algorithm for confidence scoring^90^ based on the improved Greengenes taxonomy^91^, and nontarget sequences (chloroplasts, mitochondria, and eukarya) and singletons were removed from the data set. High-quality sequences were assigned to operational taxonomic units (OTUs) in mothur based on 97% sequence identity and the average neighbor clustering algorithm, and the taxonomic classification of each OTU was determined by majority consensus^87^. To standardize sampling depths (i.e. number of sequence reads) among the different replicates, each data set was subsampled to the lowest read count (*n* = 24,595) from the final shared file, and all subsequent data analyses were based on the resulting subsampled data sets.

### Microbial community diversity analysis

To compare microbial community diversity among the four sources (three ascidian species and ambient seawater), alpha diversity metrics for OTU richness and evenness were calculated in mothur, including observed OTU richness (S), expected OTU richness (Chao1), the Simpson evenness index (E1/D), the inverse Simpson index (D) and the Shannon-Weaver diversity index (H′). Analyses of variance (ANOVA) were used to statistically compare the diversity indices among the four sources, and Tukey’s honest significance difference (HSD) tests were performed for multiple pairwise post hoc comparisons of means.

### Microbial OTU overlap and core symbiont communities

Venn diagrams were constructed in mothur to visualize OTU overlap among sampling sources. Core OTU communities were identified for each host (“host-specific” core) and across all ascidian hosts (“universal” core). Host-specific core communities included OTUs present in all replicate samples of each host species. Universal core communities included OTUs present in all replicates of all host species. Representative sequences from the 15 most abundant core OTUs (host-specific and universal) were analyzed using BLASTn to identify related sequences in GenBank and compare corresponding sources. Sequence comparisons were based on highest percent identity matches and identified sources.

### Microbial community structure analysis

To assess host-specificity and compare the structure of microbial symbiont communities among the four sources, Bray-Curtis similarity matrices were created based on OTU relative abundances in PRIMER (version 6.1.11) and visualized in multi-dimensional scaling plots. Permutational multivariate analyses of variance (PERMANOVA) were used to statistically compare the structure of microbial communities among the four sources, including multiple pairwise comparisons for significant main test PERMANOVA results. Pairwise comparisons were corrected via the Benjamini-Yekutieli (B-Y) false-discovery rate control and an experiment-wise error rate of α = 0.05^92^. Permutational multivariate analyses of dispersion (PERMDISP) were performed to verify that significant PERMANOVA results stemmed from structural differences, not unequal dispersion of variability, among groups. Sequence data were divided into abundant and rare-OTU partitions using a 0.1% relative abundance threshold^43^, resulting in a cutoff value of 24 sequences (“abundant” OTUs contained >24 sequences, “rare” OTUs contained ≤24 sequences). Bray-Curtis similarity matrices were created for each data partition and PERMANOVA were used to test for differences in the structure of abundant and rare members of the microbiome.

## Electronic supplementary material

Supplemental Materials
