# Author Correction: Introduced ascidians harbor highly diverse and host-specific symbiotic microbial assemblages

**DOI:** 10.1038/s41598-018-23758-9

**Published:** 2018-04-20

**Authors:** James S. Evans, Patrick M. Erwin, Noa Shenkar, Susanna López-Legentil

**Affiliations:** 1 Department of Biology & Marine Biology, and Center for Marine Science, University of North Carolina Wilmington, 5600 Marvin K. Moss Lane, Wilmington, NC 28409 United States of America; 2 Department of Zoology, and The Steinhardt Museum of Natural History, Israel National Center for Biodiversity Studies, Tel-Aviv University, Tel Aviv, 69978 Israel

Correction to: *Scientific Reports* 10.1038/s41598-017-11441-4, published online 08 September 2017

The original version of this Article contained an error in the bioinformatics pathway that resulted in the misalignment of OTU abundances with the incorrect corresponding OTU identities. In addition, the alignment database utilized was misreported as Greengenes instead of SILVA.

As a result, in the Abstract,

“Ascidians hosted diverse symbiont communities, consisting of 5,696 unique microbial OTUs (at 97% sequenced identity) from 47 bacterial and three archaeal phyla.”

now reads:

“Ascidians hosted diverse symbiont communities, consisting of 5,696 unique microbial OTUs (at 97% sequenced identity) from 44 bacterial and three archaeal phyla.”

In the results section under subheading ‘Symbiotic microbial community composition and diversity’,

“Ascidian-sourced OTUs spanned 42 bacterial and three archaeal phyla (Euryarchaeota, Crenarchaeota, and Parvarchaeota), with all archaeal phyla represented in all three ascidian species, though relative abundances varied among the different sources (Fig. 2a). Bacterial phyla likewise differed in relative abundance among the three species investigated (Fig. 2a). Microbial communities in D. bermudensis included 36 bacterial phyla and were dominated by Alphaproteobacteria (68%) and Gammaproteobacteria (7%), as well as Crenarchaeota (16%; Fig. 2b). Symbiont communities in P. anguinea included 37 bacterial phyla and were dominated by unclassified Bacteria (34%), Alphaproteobacteria (27%), Gammaproteobacteria (18%) and Bacteroidetes (10%; Fig. 2c). Microbial communities in P. zorritensis included 40 bacterial phyla and were dominated by Alphaproteobacteria (42%), Gammaproteobacteria (24%), Bacteroidetes (10%), and Planctomycetes (4%; Fig. 2d).”

now reads:

“Ascidian-sourced OTUs spanned 44 bacterial and three archaeal phyla (Euryarchaeota, Crenarchaeota, and Parvarchaeota), with Euryarchaeota and Crenarchaeota represented in all three ascidian species, though relative abundances varied among the different sources (Figure 2a). Parvarchaeota was detected only in the two Polyandrocarpa species, and only in low concentrations. Bacterial phyla likewise differed in relative abundance among the three species investigated (Figure 2a). Microbial communities in D. bermudensis included 32 bacterial phyla and were dominated by Alphaproteobacteria (38%), Gammaproteobacteria (22%), unclassified bacteria (19%), and Bacteroidetes (6%; Figure 2b). Symbiont communities in P. anguinea included 39 bacterial phyla and were dominated by Alphaproteobacteria (66%) and Gammaproteobacteria (7%), as well as Crenarchaeota (13%; Figure 2c). Microbial communities in P. zorritensis included 42 bacterial phyla and were dominated by Alphaproteobacteria (47%), Gammaproteobacteria (17%), Bacteroidetes (9%), and Planctomycetes (5%), as well as Crenarchaeota (9%; Figure 2d).”

In the Results section under subheading ‘Core symbiont community composition and diversity’,

“This one unclassified OTU represented 98.6% of all unclassified bacterial sequences in *P. anguinea* and 95.8% of unclassified sequences for all three species together.”

now reads:

“This one unclassified OTU represented 98.3% of all unclassified bacterial sequences in *D. bermudensis* and 94.0% of unclassified sequences for all three species together.”

In the Discussion section,

“Indeed, in the current study, most universal ascidian symbiont OTUs (80.3%) were also detected in the seawater, with three of these OTUs matching identically (100% pairwise identity) to sequences previously reported from seawater samples or sponge tissue within the same region^39^.”

now reads:

“Indeed, in the current study, most universal ascidian symbiont OTUs (80.3%) were also detected in the seawater, with some of these OTUs matching identically (100% pairwise identity) to sequences previously reported from seawater samples or sponge tissue within the same region^39^.”

In the Methods section under subheading ‘Next-generation sequence data processing’,

“Briefly, raw sequences were quality-filtered and aligned to the Greengenes reference database (gg_13_5_99), putative chimeric sequences were removed via self-reference searching with UChime^89^, sequences were classified using a naive Bayesian classifier and bootstrap algorithm for confidence scoring^90^ based on the improved Greengenes taxonomy^91^, and nontarget sequences (chloroplasts, mitochondria, and eukarya) and singletons were removed from the data set.”

now reads:

“Briefly, raw sequences were quality-filtered and aligned to the SILVA reference database (v119), putative chimeric sequences were removed via self-reference searching with UChime^89^, sequences were classified using a naive Bayesian classifier and bootstrap algorithm for confidence scoring^90^ based on the improved Greengenes taxonomy^91^, and nontarget sequences (chloroplasts, mitochondria, and eukarya) and singletons were removed from the data set.”

These errors also affected Tables 2 and 3 and Figure 2 which have all been replaced.

In the Supplementary Information file originally published with this Article, (Table S2) was inadvertently incorrectly included and has now been removed.

These errors have now been corrected in the HTML and PDF versions of this Article, and in the accompanying Supplementary Material.

