# Supplementary material for: Introduced ascidians harbor highly diverse and host-specific symbiotic microbial assemblages
Source: Sci Rep. 2017 Sep 8;7:11033. doi: 10.1038/s41598-017-11441-4 (PMC5591302; doi:10.1038/s41598-017-11441-4)
Supplement: Supplementary file 1 — Supplemental Materials [file 41598_2017_11441_MOESM1_ESM.docx]

**Introduced ascidians harbor highly diverse and host-specific symbiotic microbial assemblages**

James S. Evans^1^ · Patrick M. Erwin^1^ · Noa Shenkar^2^ · Susanna López-Legentil^1,^*

^1^*Department of Biology & Marine Biology, and Center for Marine Science, University of North Carolina Wilmington, 5600 Marvin K. Moss Lane, Wilmington NC 28409, United States of America*

*^2^Department of Zoology, and The Steinhardt Museum of Natural History, Israel National Center for Biodiversity Studies, Tel-Aviv University, Tel Aviv 69978, Israel*

*Corresponding author: Susanna López-Legentil; email: [lopezlegentils@uncw.edu](mailto:lopezlegentils@uncw.edu); Telephone: +1(910) 962-2615; Fax: +1 (910) 962-2410

**Table S1.** Diversity metrics for microbial communities associated with *Distaplia bermudensis*, *Polyandrocarpa anguinea*, and *P. zorritensis* at overall (a), abundant (b), and rare (c) data partitions. Average values (±1 SD) are shown, with different superscript letters denoting significantly different means among sources.

| **a.)** Overall (all OTUs) | | | | | | | | |
| --- | --- | --- | --- | --- | --- | --- | --- | --- |
| **Source** | **S** | **Chao 1** | | **E1/D** | | **D** | **H’** | |
| *D. bermudensis* | 715.8 ± 225.0 ^C^ | 1604.5 ± 387.1 ^C^ | | 0.010 ± 0.006 ^A^ | | 8.211 ± 7.618 ^A^ | 2.567 ± 0.976 ^B^ | |
| *P. anguinea* | 1318.6 ± 101.3 ^B^ | 2220.3± 161.7 ^B^ | | 0.004 ± 0.001 ^A^ | | 5.740 ± 1.133 ^A^ | 3.225 ± 0.202 ^AB^ | |
| *P. zorritensis* | 1970.6 ± 191.1 ^A^ | 2993.6 ± 206.8 ^A^ | | 0.007 ± 0.004 ^A^ | | 13.11 ± 9.111 ^A^ | 4.464 ± 0.833 ^A^ | |
|  | | | | | | | | |
| **b.)** Abundant OTUs | | | | | | | | |
| **Source** | **S** | | **Chao 1** | | **E1/D** | **D** | | **H’** |
| *D. bermudensis* | 363.0 ± 67.42 ^C^ | | 533.6 ± 79.61 ^B^ | | 0.020 ± 0.013 ^A^ | 7.706 ± 6.795 ^A^ | | 2.410 ± 0.903 ^B^ |
| *P. anguinea* | 549.6 ± 14.60 ^B^ | | 644.4 ± 20.77 ^A^ | | 0.009 ± 0.002 ^A^ | 5.129 ± 0.990 ^A^ | | 2.821 ± 0.168 ^AB^ |
| *P. zorritensis* | 639.2 ± 29.05 ^A^ | | 688.2 ± 30.99 ^A^ | | 0.016 ± 0.010 ^A^ | 10.25 ± 3.054 ^A^ | | 3.805 ± 0.781 ^A^ |
|  | | | | | | | | |
| **c.)** Rare OTUs | | | | | | | | |
| **Source** | **S** | | **Chao 1** | | **E1/D** | **D** | | **H’** |
| *D. bermudensis* | 352.8 ± 158.0 ^C^ | | 1342.8 ± 226.4 ^B^ | | 2.282 ± 1.370 ^A^ | 656.4 ± 258.0 ^B^ | | 5.646 ± 0.292 ^C^ |
| *P. anguinea* | 769.0 ± 89.14 ^B^ | | 1676.8 ± 176.7 ^B^ | | 0.755 ± 0.087 ^B^ | 582.1 ± 107.9 ^B^ | | 6.370 ± 0.121 ^B^ |
| *P. zorritensis* | 1331.4 ± 168.7 ^A^ | | 2392.3 ± 219.9 ^A^ | | 0.796 ± 0.209 ^B^ | 1041.1 ± 188.3 ^A^ | | 6.919 ± 0.093 ^A^ |

*S* observed richness, *Chao 1* expected richness, *E1/D* Simpson Evenness, *D* Inverse Simpson, *H’* Shannon Weaver.
